# Cancer organoid co-culture model system: Novel approach to guide precision medicine

**DOI:** 10.3389/fimmu.2022.1061388

**Published:** 2023-01-12

**Authors:** Jin Yuan, Xiaoyang Li, Shengji Yu

**Affiliations:** Department of Orthopedics, National Cancer Center/National Clinical Research Center for Cancer/Cancer Hospital, Chinese Academy of Medical Sciences and Peking Union Medical College, Beijing, China

**Keywords:** cancer organoids, co-culture models, tumor microenvironment, cancer-associated fibroblasts, CAR-T cells

## Abstract

Three-dimensional cancer organoids derived from self-organizing cancer stems are ex vivo miniatures of tumors that faithfully recapitulate their structure, distinctive cancer features, and genetic signatures. As novel tools, current cancer organoids have been well established and rapidly applied in drug testing, genome editing, and transplantation, with the ultimate aim of entering clinical practice for guiding personalized therapy. However, given that the lack of a tumor microenvironment, including immune cells and fibrous cells, is a major limitation of this emerging methodology, co-culture models inspire high hope for further application of this technology in cancer research. Co-culture of cancer organoids and immune cells or fibroblasts is available to investigate the tumor microenvironment, molecular interactions, and chimeric antigen receptor-engineered lymphocytes in cancer treatment. In light of the recent progress in cancer organoid co-culture models, it is only possible to recognize the advantages and drawbacks of this novel model to exploit its full potential. In this review, we summarize the recent advances in the application of cancer organoids and co-culture models and how they could be improved in the future to benefit cancer research, especially precision medicine.

## 1 Introduction

Cancer precision medicine has been deeply and widely explored through experimental studies to clinical evaluations to achieve the goal of individualized treatment and improve patient prognosis. However, genotype-based cancer precision medicine has some limitations or deficiencies. Genomic instability during sustained cancer proliferation within the tumor microenvironment (TME) gives rise to intra- and inter-tumoral heterogeneity, contributing to drug resistance, treatment failure, and progression. Cancer development is an evolving, highly regulated, dynamic biochemical process associated with TME. Genetic mutations accumulated in the copying process during DNA replication of cancer cells drive the generation of intratumoral heterogeneity within a tumor or inter-tumoral heterogeneity between tumors, with interactions among cancer cells, immune cells, and fibroblasts leading to TME alterations. Moreover, tumor heterogeneity increases due to TME alterations. Thus, the provoked interaction network within TME hinders the clinical application of precision cancer therapy. Further developing preclinical models to investigate the TME and guide clinical precision therapy is required.

Organoids are three-dimensional (3D) cell clusters *in vitro* that contain key characteristics of an organ *in vivo*, including a self-organizing stem cell population that can differentiate into organ-specific cells (1). Organoids recapitulate the structure, function, and genetic signature of the original organ, thus providing a solid foundation for future research on stem cells, regenerative biology, organogenesis, precision medicine, and human pathologies (2). Organoids can be derived from embryonic stem cells (ESCs), induced pluripotent stem cells (iPSCs), and adult stem cells (ASCs) through a process similar to the acquisition of their distinctive organization (1). Self-assembly and differentiation through organoid formation depend on implicated cell signaling pathways mediated by intrinsic components and the extracellular environment, including the extracellular matrix (ECM) and media. Due to a better understanding of ECM biology and technologies for cell culture (3, 4), organoid culture has been progressively implemented. Moreover, organoids have shown exciting potential and have been a popular research focus in tissue engineering and biological research in the past decade.

Intra-tumoral heterogeneity is one of the characteristics of cancers that promotes tumor evolution and makes cancer treatment challenging (5). Given that heterogeneous cancer cells and cancer-associated cells within a tumor contribute unequally to progression with complex intercellular interactions, cancer research findings using 2D cancer cell lines have been challenged. Cancer organoids retain the 3D structure of the TME, providing a physical context for molecular interactions. Recently, a cancer organoid co-culture model was developed and utilized to elucidate cell-to-cell interactions, mechanisms of cancer immune responses, and the mechanism of tumor metastasis. We summarize the history of cancer organoids that show great potential and promise in modeling human diseases. We also summarized the applications of cancer organoids in cancer research on multiple systems in the human body. Various excellent reviews have discussed the applications of cancer organoid model systems in genomic analysis and drug screening. Here, we discuss a novel cancer organoid co-culture model system with the advancement of its applications for investigating cell-cell interactions, immune response within cancers, and underlying mechanisms of cancer evolution and personalized precision medicine. We also provide further insights into novel applications and development directions of the cancer organoid co-culture model system. We hope that this review provides a better perspective to help researchers apply this practical method to cancer research and precision medicine.

## 2 Organoid overview

**Figure 1:**
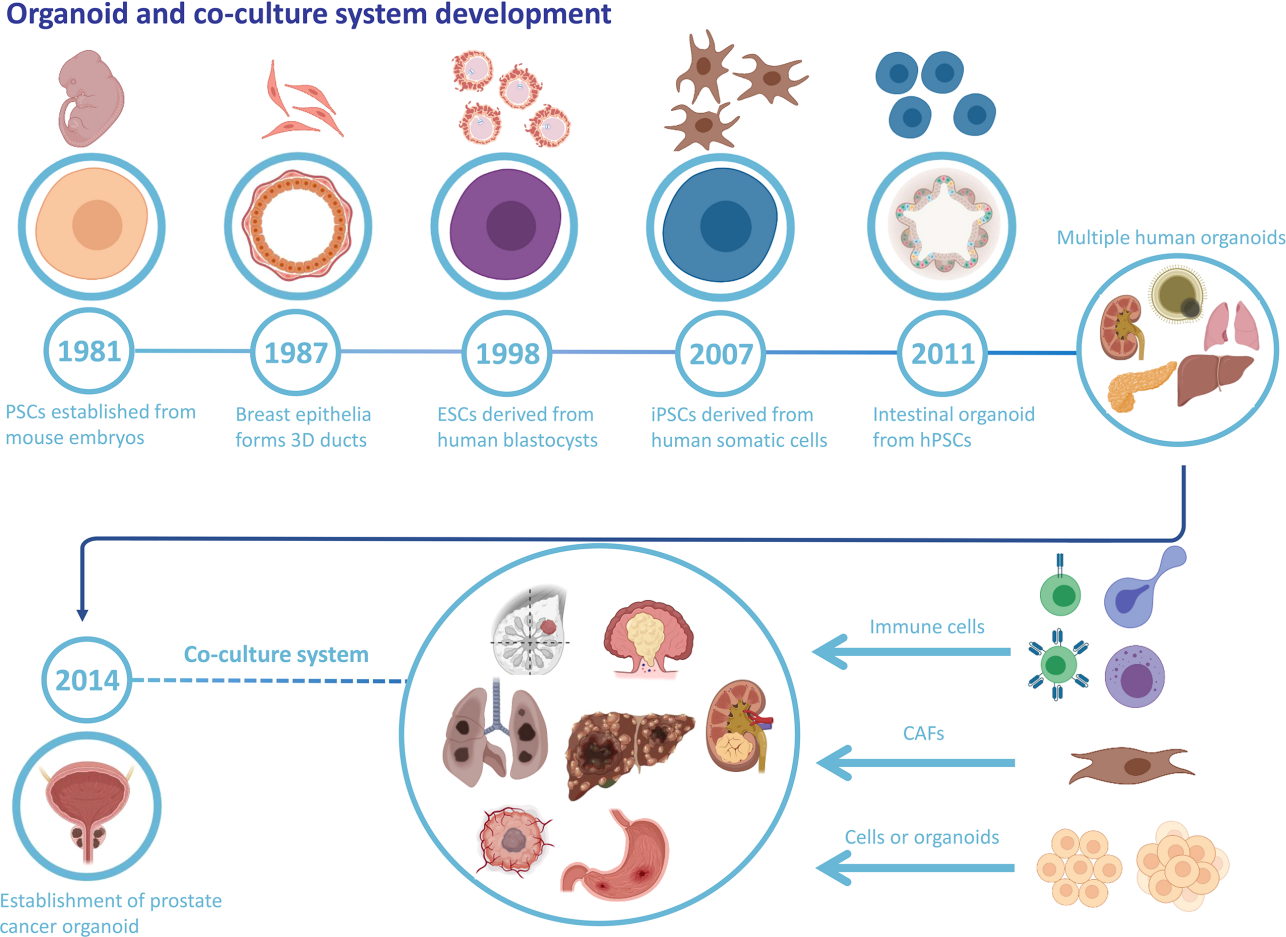
Organoid and co-culture system development. The history of organoid and cancer organoids and the well-established co-culture system of various cancer organoids and specific cell types.

An organoid is a stem cell-derived 3D cell culture that obtains the structure, collection of multiple cell types, and functionality of the corresponding tissue. Organoids are promising candidates for biomedical applications with tremendous potential and appealing prospects (6, 7). For the past few decades, numerous studies of stem cells have led to a better understanding of their behavior, with emerging methodologies of controlling stem cells’ self-organization and differentiation, which provides a scientific basis for the further establishment of organoids (7). There are two types of stem cells. One is ESCs and the other is ASCs. ESCs possess developmental totipotency and can differentiate into all cell types. ASCs are undifferentiated stem cells with the capacity to differentiate, maintain homeostasis, and regenerate in a specific organ (8). Since 1981, substantial scientific progress in stem cell research has paved the way for organoid development (**Figure 1**). Moreover, more restricted ASCs have exhibited the capacity to form organoids *in vitro* once grown in the appropriate extracellular matrix and provided with specific molecular factors (9). The critical point for organoid formation and growth is whether the culture conditions duplicate the *in vivo* niche signaling pathways for stem cells, which contributes to sustaining stem cell functions and inducing differentiation (10).

As a novel tool and methodology, organoids have been sufficiently developed, widely studied, and adopted in various biomedical research fields (11). Organoids preserve the principal features of organ biology with greater experimental accessibility than that of animal models. Moreover, studies focusing on human embryonic and fetal tissues can be easier to conduct without restricting ethical concerns, such as prenatal development and tissue maintenance. Organoids also provide a platform to simulate the pathological environment for experiments at the organ level that cannot be performed at molecular, cellular, or animal levels. Based on this, precision medicine can be carried out using an organoid-based high-throughput screening and profiling strategy, facilitating preclinical evaluation and treatment guidance.

## 3 Cancer organoid

Organoids have been widely used to investigate neoplastic diseases, aside from being used to establish a normal developmental model. Patient-derived prostate cancer organoids were established for nearly a decade by Chen et al. (12). Recently, various cancer organoids, including colorectal cancer (CRC) (13), breast cancer (14), hepatocellular cancer (15), and non-small cell lung cancer (16), have been developed for drug screening, radiotherapy screening, genome editing, transplantation, and oncogene identification (**Table 1**). Most cancer organoids were obtained from patient-derived cancer samples and generated under ASCs-organoid conditions, but CRISPR-Cas9 nuclease genome editing system established the minority. Yilmaz et al. established CRC organoids *via* CRISPR-Cas9-based APC editing with lentivirus transfection using colon organoids derived from transgenic mice (33). Few studies have focused on developing organoids using other biological samples. Gao et al. successfully established prostate cancer organoids by culturing collected circulating tumor cells (12). Moreover, cancer organoids from cells in the urine and bronchoalveolar lavage fluid have already been established, providing a novel approach for cancer organoid establishment (34, 35).

**Table 1:** Applications of organoids in cancers.

	Cancer type	Source	Establishment success rate	Organoid models	Max passage number	Drug screening	Radiotherapy screening	Genome editing and transplantation	Related genes and molecular activity	Ref.
Digestive system	CRC	Biopsy	63%(40/63)	PDO	N	√	×	×	Prediction of drug response; irinotecan; 5-FU; 5-FU–oxaliplatin; combination therapy	(13)
	HCC	Surgery	50%	PDO	N	√	×	×	Hedgehog inhibition, sorafenib resistance; CD44	(15)
	GC	Surgery	N	PDO	N	√	×	×	KHDRBS3, CD44, 5-FU	(17)
	GC	Surgery	N	PDO	4	√	×	√	Epirubicin, oxaliplatin, 5-FU	(18)
	PDAC	N	N	PDO	25	√	×	×	IL-1, JAK/STAT signaling, TGF-β, NF-κB	(19)
	ESCC	Biopsy	71.4%(15/21)	PDO	7	×	×	×	Autophagy, CD44, 5-Fluorouracil	(20)
Respiratory system	NSCLC	Surgery	88%(57/65)	PDO, PDXO	≥10	√	×	×	Mutation and copy number landscape; characterization (growth, purity, histologic/lineage marker)	(16)
	LADC	Surgery	80%(12/15)	PDO	≥10	√	×	×	RHOF, SLC16A3, ANXA10, CDHR1	(21)
Genital system	OC	Surgery	N	PDO	N	√	×	×	TME; AKT2, KRAS, CCNE1	(22)
	EC	Biopsy	N	PDO	6	√	×	×	Precancer pathologies, PTEN, CTCF, ARID1A, PIK3CA, TP53, ARID1A, POLE, FAT1, CTNNB1	(23)
	cCCC	Biopsy	N	PDO	7	√	×	×	MLH1, TFE3, PARP1, FANCD2, PMS2, MET	(24)
Central nerve system	GBM	Surgery	91.4%; 66.7%; 75%	PDO	N	√	×	×	EGFR variant III	(25)
	Ms	Surgery	N	PDO	N	√	√	×	FOXM1 inhibition; tumor proliferation	(26)
	MB	N	N	PDO	N	√	×	×	Otx2, c-MYC; SMARCA4; EZH2 inhibition	(27)
Urinary system	UBC	Surgery	70%(12/17)	PDO	26	√	×	×	Clonal evolution; treatment response	(28)
	PCa	Surgery, biopsy	16%(4/25)	PDO, PDOX	35	√	×	×	EZH2 inhibition; drug screening	(29)
Endocrine system	PTC	Biopsy	7%	PDO	5	×	√	×	Early diagnosis of non-responding patients	(30)
Breast	BC	N	N	–	20	×	×	√	Modeling Breast Cancer, knockout of P53, PTEN, RB1, NF1	(14)
	BC	Biopsy	N	PDO	4	√	×	×	Representative of the tissue origin in primary culture	(31)
Mixed	TME	Surgery	73%	PDO, PDXO	4	×	×	×	Preserved the original tumor T cell receptor spectrum	(32)

N: not mentioned; √, yes; ×, no.

BC, breast cancer; cCCC, cervical clear cell carcinoma; CRC, colorectal cancer; EC, endometrial cancer; ESCC, esophageal squamous carcinoma; GBM, glioblastoma; GC, gastric cancer; HCC, hepatocellular carcinoma; LADC, lung adenocarcinoma; MB, medulloblastoma; Ms, Meningiomas; NSCLC, non-small-cell lung cancer; PCa, prostate cancer; PDAC, pancreatic ductal adenocarcinoma; PTC, papillary thyroid cancer; OC, ovarian cancer; TME, tumor microenvironment; UBC, urinary bladder cancer.

Likewise, cancer organoids contain the cancerous cell composition of the original tumors and possess the corresponding features and genetics. The generation of various cancer organoids requires distinct methods. There are no standardized culture media or procedures for experiments. Optimal tissues are commonly obtained from tumor margins with a minimal necrosis rate. Generally, the entire process is initiated by mechanical and enzymatic digestion of tumor samples into ~1 mm diameter pieces, subsequently seeding the tissue suspension onto Matrigel as a biomimetic scaffold. Matrigel, which mainly contains laminin, entactin, proteoglycans, and collagen IV, primarily contributes to the cellular architecture of organoids (36). Unlike culturing healthy organoids, media with reduced growth factors is preferred for cancer organoid culture to minimize clonal selection and avoid confounding drug treatment effects (37). Growth factors for cancer organoid culture include Wnt3A, R-spondin-1, TGF-β receptor inhibitor, epidermal growth factor, and Noggin, but the combination and concentration of these factors added to the media depend on the specific cancer type. Compared to the initiated culture process, organoid passage is a more simplified but essential process during the culture period. The issue we must be concerned with is the passage number of cancer organoids that can be used for investigations. Upon passage, the genetic material of cancer cells within organoids undergoes mutations or alterations (18, 20, 23). Consequently, the authentication of cancer organoids is particularly important for their authenticity and credibility. Although cancer organoids are preferred and widely used in cancer research, their limitations cannot be neglected. Exogenous growth factors and small molecules added to organoid growth may result in clonal selection, and the components of the culture media may interact with the tested drug, complicating the conclusion. In addition, inappropriate sample collection may significantly impact the successful culturing rate because of less active proliferating cells and increased necrosis.

## 4 Cancer organoid application

**Figure 2:**
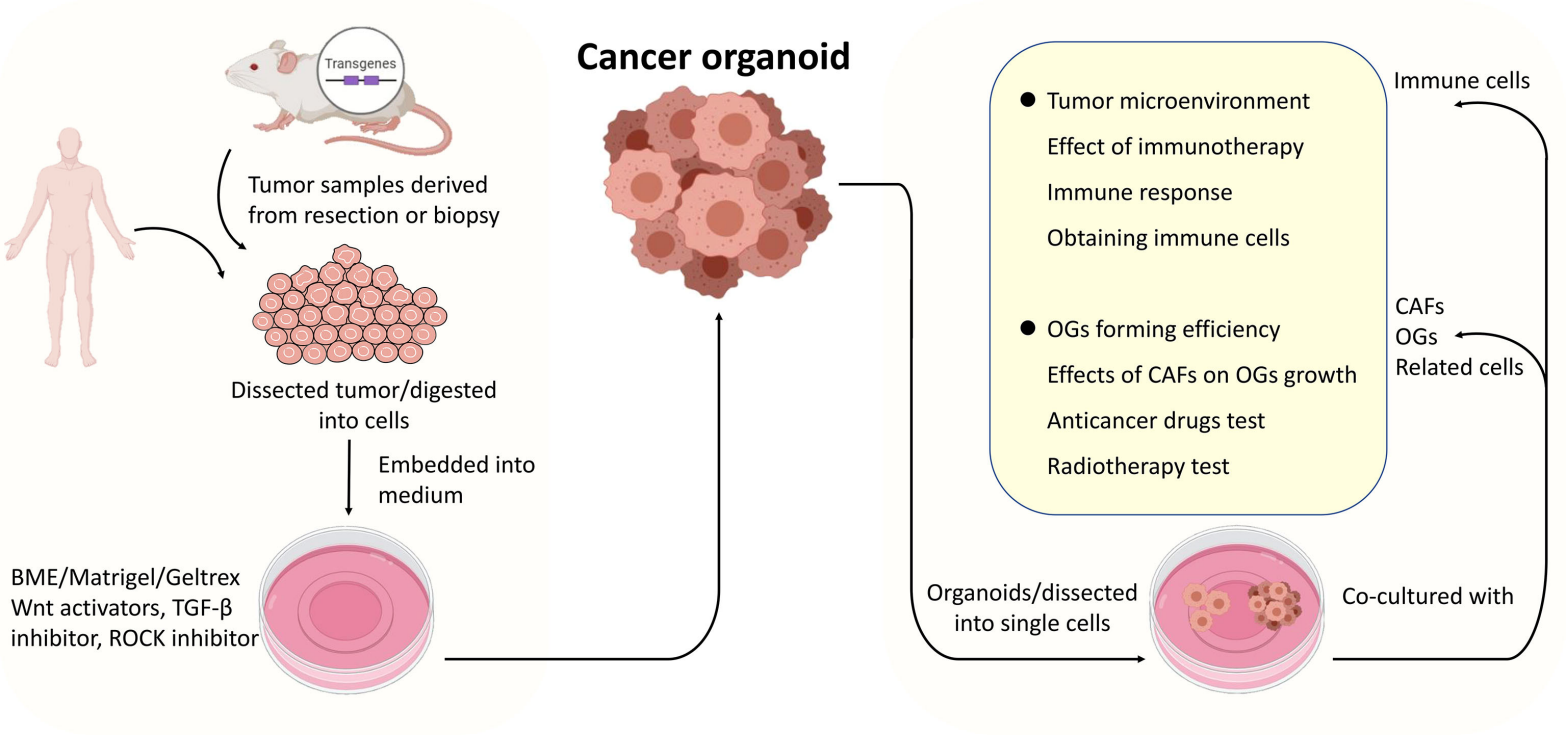
The procedures of cancer organoids establishment and applications of the co-culture system. Tumor tissues derived from surgically resected tumors or biopsies were dissected into small pieces or digested into cells, mixed with Matrigel, and cultured in media supplemented with specific growth factors. Cancer organoids (dissected into cells or not) were co-cultured with organoids, cancer-associated fibroblasts or related cells primarily immune cells to model the interactions between specific cells within tumors or TME.

As advancements have been made in cancer organoid development, cancer organoids have become a widely accepted practical model in cancer research (**Figure 2**). Cancer organoids are primarily used in drug screening for personalized medicine approaches. The drug response of patient-derived organoids (PDOs) mainly simulates patients’ initial responses to the same agents (28, 38–40). These studies have shown that the genetic changes that drive oncogenic pathways correspond to this therapy. Therefore, drug screening is the primary part of cancer organoid applications in cancer research to screen the most effective drugs and predict their therapeutic effects. A combination of other conditions, including radiation, was added to determine the most effective therapeutic strategy. For example, the FOXM1 inhibitor thiostrepton combined with radiotherapy (4 Gy) remarkably suppresses the proliferation of meningioma organoid models (26). Such a cancer organoid model may be applied in immunoradiotherapy investigations. In addition, the transplantation model is an excellent platform for mimicking human disease.

The orthotopic transplantation of cancer organoids has been established in preclinical models. Sequencing analysis is commonly used to identify type-specific differentially expressed genes. RNA sequencing analysis and whole-exome sequencing were performed to identify cancer-related oncogenes. Moreover, as time passes, these techniques are the primary authentication methods for cancer organoids, and targeted sequencing is used to identify target mutations. CRISPR-Cas9 technology is revolutionary in genome editing and has been applied in organoid research. However, the precise integration of exogenous DNA sequences into human organoids is deficient in knock-in approaches. To address this, CRISPR-Cas9-mediated homology-independent organoid transgenesis was established to enable the efficient generation of knock-in human organoids representing different tissues (41), concluding that this technique can be used to achieve fast and efficient gene knock-in in human wild-type organoids.

Cancer organoids are now versatile tools in cancer research with a wide range of potential applications, showing the prospects of this advanced methodology in cancer research. However, there are some shortcomings. Organoids only comprise the epithelial layer without the native microenvironment of the surrounding mesenchyme, immune cells, nervous system, or muscular layer (42). Developing a novel co-culture model system of cancer organoids with other cells or organoids may recapitulate cell-cell interactions. In addition, the culture media should be improved to promote organoid growth and long-term expansion while minimizing the impact of growth factors in the media on the behavior of organoids (43). Cancer organoids have been established based on various human cancers; however, some cancer types, such as sarcomas, are still not involved. Consequently, refining culture approaches for rare heterogeneous cancer organoids and applying this tool for precision medicine should be addressed in future studies.

## 5 Co-culture model in cancer organoids

Co-culture is a method for culturing multiple distinct cell types, directly or indirectly, within the same culture environment (44). The cancer organoid co-culture model can efficiently simulate the environment for interactions between cancer organoids and cells within a tumor. Given that there are no immune cells, the nervous system, or mature TME in cancer organoids, a co-culture model was developed to solve this problem. The cancer organoid co-culture model was established for three main purposes. The first and most common application is to drive organoid formation *via* direct or indirect interactions between specific cell types within tumors. The second is the generation of specific tumor-targeting cytotoxic immune cells for cancer therapy using cancer organoids. The third is to detect the immune crosstalk between cancer organoids and specific cells, which is commonly implemented as a suspension of one cell population, usually cancer-associated fibroblasts, with the secretion of signaling factors and cytokines to condition the medium for the organoid. Marked advances in cancer organoids have been made by employing co-cultures of cancer organoids with specific cell types (**Table 2**). Moreover, the cancer organoid co-culture system with a specific type of cell can be used for different research purposes, and the co-culture of cancer organoids with multiple types of cells may accurately mimic tumor conditions.

**Table 2:** Co-culture modeling systems of cancer OGs with biological subjects.

Co-cultures			Cancer OGs	Ratio	Time	Additional condition	Single cells	Modeling for	Related genes/molecules	Year	References
Cells	Immune cells	CTLs/dendritic cells*	GC*	–	16 h	–	No	Determination of efficacy of immune checkpoint inhibition on cancer cell growth in an in vitro model	PD-1	2018	(45)
		Lymphocytes	PDAC	–	72 h	–	No	Establishment of PDAC TME	–	2018	(46)
		Lymphocytes	Mixed	20:1	7 d	IL-2 and anti-PD-1	Yes	Obtain tumor reactive T cells	IFNγ, IL-2, PD-L1	2018	(47)
		CTLs/dendritic cells	PDAC	–	96 h	–	No	Determination of the effect of inhibition of PD-L1/PD-1 interaction and PMN-MDSC depletion on PDAC	ARG1, NOS2, NO	2020	(48)
		Macrophage*	CRC*	–	48 h	–	Yes	Establishment of CRC TME	P38, TGF-β/BMP, MAPK, PI3K, NF-κB	2020	(49)
		NK cells	PDAC	–	7-14d	–	No	Identification of impact of PDAC on NK phenotype	PVR, MICA, ULBP2, IL-10	2020	(50)
		CD8^+^/dendritic cells	GC	250:1	–	–	No	Prediction of CD8^+^ cells therapy using tumor-antigen presented DCs to expand CD8^+^ T cells	PD-L1	2021	(51)
		op-T cells	PDAC	1:1	7 d	–	No	Generation of CD8^+^ or CD4^+^opT cells and test of tumor-killing efficacy	PD-L1 or TIM3 or TIGIT or LAG3	2021	(52)
		CTL/ MDSCs	GC	–	48 h	Nivolumab, Mubritinib, Cabozantinib	No	Determination of concomitant effect of HER2 and PD-L1 and screen responses to a combination of anti-HER2 and immunotherapy	AKT/mTOR, PD-L1, HER2	2021	(53)
		Macrophages/CD8^+^ T cells	CRC	2/1:2	24 h	–	No	Determination of the relationships among immune cells and tumor cells	SIRT1, CXCR4/CXCL12	2022	(54)
		NK cells	BC	30:1	–	–	No	Test direct anti-tumor cytotoxicity and antibody-dependent cell-mediated cytotoxicity	–	2022	(55)
		CD3^+^ T cells	CCA	25-50:1	7 d	IL-2	Yes	Test anti-tumor organoid immune response	Immune checkpoint expression, CYFRA	2022	(56)
	CAFs										
			LUSC	2:1	8-12 d	–	Yes	Capture key components of the TME	SOX2	2018	(57)
			CRC	–	–	-、	No	Determination of fibroblasts on tumor growth and malignancy	Wnt, Sfrp1, Dkk1, E-cadherin, Zeb1, Vim, Ctnnb1	2020	(58)
			CRC	between 2:1 and 3:1	–	Capecitabine, 5-FU, oxaliplatin and irinotecan	No	Investigation of the interaction of CRC with CAFs	PI3K-Akt	2020	(59)
			OSCC	1:1	–	–	No	Determination of OGs forming efficiency	Lactate, CD44+, OXPHOS, MCT1	2021	(60)
			HCC/CCA	1:1	–	Sorafenib, regorafenib, 5-FU	Yes	Investigation of interactions of CCA and CAFs; examination of the effects of CAFs on the response of OGs to the anticancer drugs	FAP, CD29, Periostin, IL6, IL17A, IGF1, IGF2, NO	2021	(61)
			OSCC	1:1	7-10 d	–	Yes	Determination of OGs forming efficiency and phenotype transition of paracancerous fibroblasts	Notch	2021	(62)
			PDAC	10:1	48 h	Gemcitabine	Yes	Determination of effect of CAFs on drug-resistance of tumor	–	2022	(63)
	PSCs		PDAC	–	–	–	Yes	Studying the phenotype of individual cell types in a mixed cell population mixed cell population	pyruvate carboxylase, malic enzyme 1	2020	(64)
	BM-MSC		CRC	1:1, 2:1, 4:1, and 8:1	–	Ultraviolet radiation and X-rays	Yes	Investigation of the interaction of CRC cells with MSCs	TNF-a, IFN-γ, PI3K/AKT, GM-CSF, CD154	2018	(65)
	FTMSC, HUVEC		FTEC	10:7:2	7 d	DKK1	Yes	Recapitulation of the early organogenesis of the fallopian tube	PAX8, LGR5, FOXJ1, Wnt	2020	(66)
	Lung epithelial cells/stromal cells		BC	–	14 d	–	Yes	Determination of Organoid-forming efficiency	Cancer-associated parenchymal cells	2019	(67)
OGs	eCOs		GBM	–	48 h	–	Yes	Determination of eCOs infiltration by GBM compared with NP spheroids	VIMENTIN, MMP2, NESTIN, SOX2	2018	(68)

*Represents the organoids were derived from mouse.

ARG1, arginase 1; BM-MSC, bone marrow-derived mesenchymal stromal cell; CAFs, Cancer-associated fibroblasts; CCA, Cholangiocarcinoma; CRC, colorectal cancer; CTLs, Cytotoxic T Lymphocyte Cells; Dkk1, Dickkopf-related protein 1; eCOs, early-stage cerebral organoids; FTEC, Fallopian tube epithelial cells; FTMSC, Fallopian tube mesenchymal stem cells; GBM, Glioblastoma; GM-CSF, Granulocyte macrophage colony-stimulating factor; HCC, Hepatocellular carcinoma; HUVEC, human umbilical vein endothelial cells; LUSC, lung squamous carcinoma; NOS2, nitric oxide synthase 2; OG, organoid; op-T cells, organoid-primed T cells; OSCC, Oral squamous cell carcinoma; PDAC, Pancreatic ductal adenocarcinoma; PMN-MDSC, Polymorphonuclear MDSC Myeloid-Derived Suppressor Cell; PSCs, Pancreatic stellate cells; ROS, reactive oxygen species; Sfrp1, Secreted frizzled-related protein 1; TME, tumor microenvironment.

### 5.1 Cancer-associated fibroblasts (CAFs) co-culture

CAFs play a major role in tumor-stromal crosstalk, which can be mediated by cell-cell contact, soluble factors, extracellular vesicles, and metabolites (69). CAFs account for most TME, particularly in CRC, and play critical roles in cancer progression, from the regulation of cancer cell proliferation and stem cell maintenance to drug resistance (70). CRC organoids and CAFs co-culture were established by Farin et al. to obtain an *in vitro* model for fibroblast plasticity in CRC, revealing that co-culture increased the contractility of CAFs, which was modulated by Wnt and IWP-2. Moreover, CRC PDO-CAF models were developed for drug testing and elucidation of CRC-CAF crosstalk, demonstrating that CAFs maintained the proliferation of CRC organoids in the hydrogels without adding growth factors and regained distinct signaling pathways that were absent in the CRC organoid culture alone but existed in tumors (59). This indicated that the CRC-CAF co-culture model was appropriate for evaluating drugs and helped bring us closer to the goal of personalized cancer medicine. Similarly, CAFs promoted the growth of *in vitro* HCC tumor organoids and transplantation xenograft models, conferring drug resistance to sorafenib, regorafenib, and 5-fluorouracil (61). This study was also conducted recently to determine the effect of CAFs on drug resistance in pancreatic ductal adenocarcinoma (PDAC) (63). CAFs are essential for tumor progression, and co-culture of CAFs and cancer organoids can recapitulate the TME within the origin of the tumor. Thus, elucidating the interactions between tumor organoids and CAFs may help improve culture media for better growth of organoids and simulation of the tumor.

Cancer organoid co-culture systems have been established for a wide range of applications. Fallopian tube epithelial cells co-cultured with fallopian stromal cells and endothelial cells form a miniature 3D structure with high efficiency, significantly suppressing Wnt inhibitors (66). Bone marrow stromal cells (BMSCs) enhance the anticancer effect of radiotherapy on CRC cells by secreting cytokines that inhibit proliferation and induce apoptosis of CRC cells. Such a mechanism would presumably be based on suppression of the PI3K/AKT signaling pathway, which may contribute to the attenuation of cell proliferation and death under irradiation with co-cultured BMSCs (65). A cancer organoid co-culture system was also developed to establish metastasis models to investigate the metastatic features of cancer. Breast cancer cells were co-cultured with lung epithelial cells to test organoid-forming efficiency (67). The cancer organoid co-culture model is an ideal tool to test the effect of various stromal cells on cancer development and progression, metastasis, and drug efficacy.

### 5.2 Immune cells co-culture

The major application of cancer organoid co-culture models is the co-culture of cancer organoids with immune cells, including cytotoxic T lymphocytes and dendritic cells (45), NK cells (55), macrophages (49, 54), and lymphocytes (46, 47) (**Table 2**). James et al. co-cultured PDAC organoids with CAFs and CD3^+^ T lymphocytes to develop a specific TME for PDAC (46). To obtain tumor-reactive T cells, peripheral blood lymphocytes were added and co-cultured with non-small cell lung cancer organoids, providing a clinically feasible strategy for generating patient-specific T cells for adoptive T cell transfer (47). Similarly, to investigate how CRC driver mutations dysregulate epithelial signaling from stromal and immune cells, CRC organoids were cultured either alone or with colonic fibroblasts and macrophages to directly compare mutation- and microenvironment-driven cell-type-specific signaling networks in CRC organoid mono- and co-cultures (49). More recently, a novel co-culture approach was developed to predict the efficacy of precision medicine to achieve a better prognosis for gastric cancer patients, using tumor antigens to stimulate antigen-presenting dendritic cells (DCs), followed by co-culture with CD8^+^ T cells to promote cytolysis and proliferation of these T cells before co-culture with patient-derived gastric cancer organoids (51). Such an approach may be considered more relevant within the TME instead of being induced by an artificial approach to T cell activation (32).

The era of checkpoint blockade immunotherapy is in full swing, exhibiting outstanding efficacy by unblocking negatively controlled T cells and triggering anticancer T cell responses. Co-culturing PDO with immune cells combined with checkpoint blockade inhibitors has been applied in a series of studies on cancer precision medicine (51–53, 56), providing important insights into predicting precision therapy efficacy with PDO. This process takes nearly ten days and includes cancer organoid establishment after surgical resection, preparation of immune cells, co-culture, drug testing, and determination of efficacy. This novel platform may guide clinical treatment at the microscopic level and benefit patients without needing long waiting periods based on advances in organoid culture techniques. Moreover, several studies have focused on generating tumor-specific lymphocytes for tumor-targeting therapy through co-culture (45–48, 51, 52). This method, with its excellent prospects, may achieve more accurate cell therapy with better clinical efficacy.

### 5.3 CAR-T cells co-culture

Currently, immunotherapy approaches, primarily immune checkpoint blockades, only select specific patient populations. The co-culture of cancer organoids and TME cells may provide an environment for immunotherapy research, making it a highly appealing and efficient option (47, 71). CAR-T cells are autologous and allogeneic T cells engineered to target specific antigens and markers on cancer cells, explicitly recognizing and eliminating cancer cells through direct T-cell cytotoxicity (72). The co-culture of cancer organoids and CAR-T cells provides a platform for predicting CAR-T cell efficacy and toxicity assessment (**Table 3**). Recently, Chen et al. established a successful preclinical testing *ex vivo* technological platform for a co-culture model system to evaluate CAR-T cell-mediated cytotoxicity against bladder cancer organoids targeting MUC1 (74). Such co-culture model systems for autologous HBVs^+^ HCC organoids and CD39^+^ HBV-CAR-T cells or CD39^+^ personalized tumor-reactive CD8^+^ T cells were also modeled to assess their anticancer efficiency (75). In addition, a co-culture of GBM organoids expressing EGFRvIII with 2173BBz CAR-T cells was performed, demonstrating the utility of rapid testing of antigen-specific CAR-T cell treatment responses (25). In addition, Farin et al. co-cultured CRC organoids with EGFRvIII-CAR NK-92 cells to model a platform for identifying and selecting suitable target antigens and assessing the anticancer activity of CAR-NK-92 cells (73). Notably, only one clinical trial has been carried out to evaluate the anticancer effects of CAR-engineered lymphocytes on cancer organoids, focusing on investigating the anticancer effects of CAR-macrophages in organoids derived from patients with breast cancer (NCT05007379). Because cancers exhibit diverse heterogeneity and genetic instability, cancer therapeutic approaches should be personalized. Cancer organoids preserve the histological features, cellular diversity, genetic heterogeneity, and mutational diversity of the tumor origin. Accordingly, co-culture of CAR-derived cells and cancer organoids can fully capture the molecular and cellular processes of immunotherapy, showing enormous potential in predicting therapeutic efficacy and cytotoxicity.

**Table 3:** Co-culture modeling systems of cancer OGs with CAR-engineered lymphocytes.

Cancer type	Colorectal cancer (73)	Glioblastoma (25)	Bladder cancer (74)	Hepatocellular carcinoma (75)
Year	2019	2020	2021	2021
Lymphocytes	NK cell line NK-92	T cells	CD4^+^, CD8^+^ T cells	CD8^+^ T cells
Antigen	EPCAM, EGFRvIII	EGFRvIII	MUC1	HBV surface protein, FRIZZLED, EPCAM
Ratio	–	–	–	10:1
Co-culture time	–	3 days	–	24 hours
Cytotoxicity assays	Luciferase-based 3D assay, live-cell imaging experiments	Cytokine ELISA, immunostaining	Cytokine detection assays	Imaging-based analysis

EGFRvIII, Epidermal growth factor receptor variant III; HBV, hepatitis B virus.

## 6 Discussion

As a major technological breakthrough, organoids are now well-established and vigorously developed as a vital methodology in biomedical studies. Organoids have been applied in tissue engineering, regenerative medicine, disease modeling, drug screening, and toxicological studies, restoring the 3D structure and primary cell types, but also in translational applications such as the prediction of chemotherapy, radiotherapy resistance before treatment, and gene editing, enabling mutation rectification (76). Although organoids have a wide range of applications in cancer research and clinical practice, the current version is a rough model, and culture procedures for specific cancer types must be constantly standardized and improved. Organoids are found in various organs that have been created, such as the brain, retina, gastrointestinal tract, tongue, thyroid, liver, pancreas, skin, lung, kidney, and heart (7). However, obstacles impede the generation of organoids in some organs, such as bone and soft tissue engineering. Human‐periosteum‐derived cells allow the scalable generation of semiautonomous callus organoids that induce the formation of bone micro-organs upon implantation (77). For most cancers, 3D organoid models have already been established for cancer investigations with barely any technological restrictions. However, no relevant studies have investigated organoids in rare malignant tumors that are equally promising in research on these rare cancers, such as bone and soft tissue sarcomas and neuroendocrine tumors. Bone and soft tissue sarcomas are commonly characterized by chromosomal translocations with a low mutational burden. Because of the advantage of gene editing of cancer organoids, mechanism-based sarcoma organoid models may provide theoretical support for investigations focusing on elucidating sarcomagenesis and other extensive applications.

Tumor cell lines in mice and patient-derived xenografts have long been used as cancer research models and have made significant contributions. However, various shortcomings hinder using these experimental models in clinical applications. Cell lines generally contain only one type of cell without co-cultured immune cells, stromal cells, TME, or organ-specific capability, losing the genetic heterogeneity of the origin tumor after multiple passages and clonal selection. In addition, there is a lack of immune response between the original tumor and the immune environment in immunodeficient mice. Human-derived cancer cells have evolved, potentially impacting chemotherapy by reshaping the genomic landscape. Such xenograft models are highly time- and resource-intensive. Cancer organoids may also overcome the aforementioned restrictions. Genetic modification of the insertions of oncogenic mutations in stem cells leads to the generation of genetically modified organoids. Unlike patient-derived xenografts, cancer organoids are easier to obtain and establish biobanks for restoration and can be used for high-throughput drug screening. Cancer organoids based on specific cancers and even on a specific individual used in high-throughput screening are expected to become powerful tools for precision therapy. Furthermore, screening can be performed using biobanks to identify new drugs and explore new indications.

**Figure 3:**
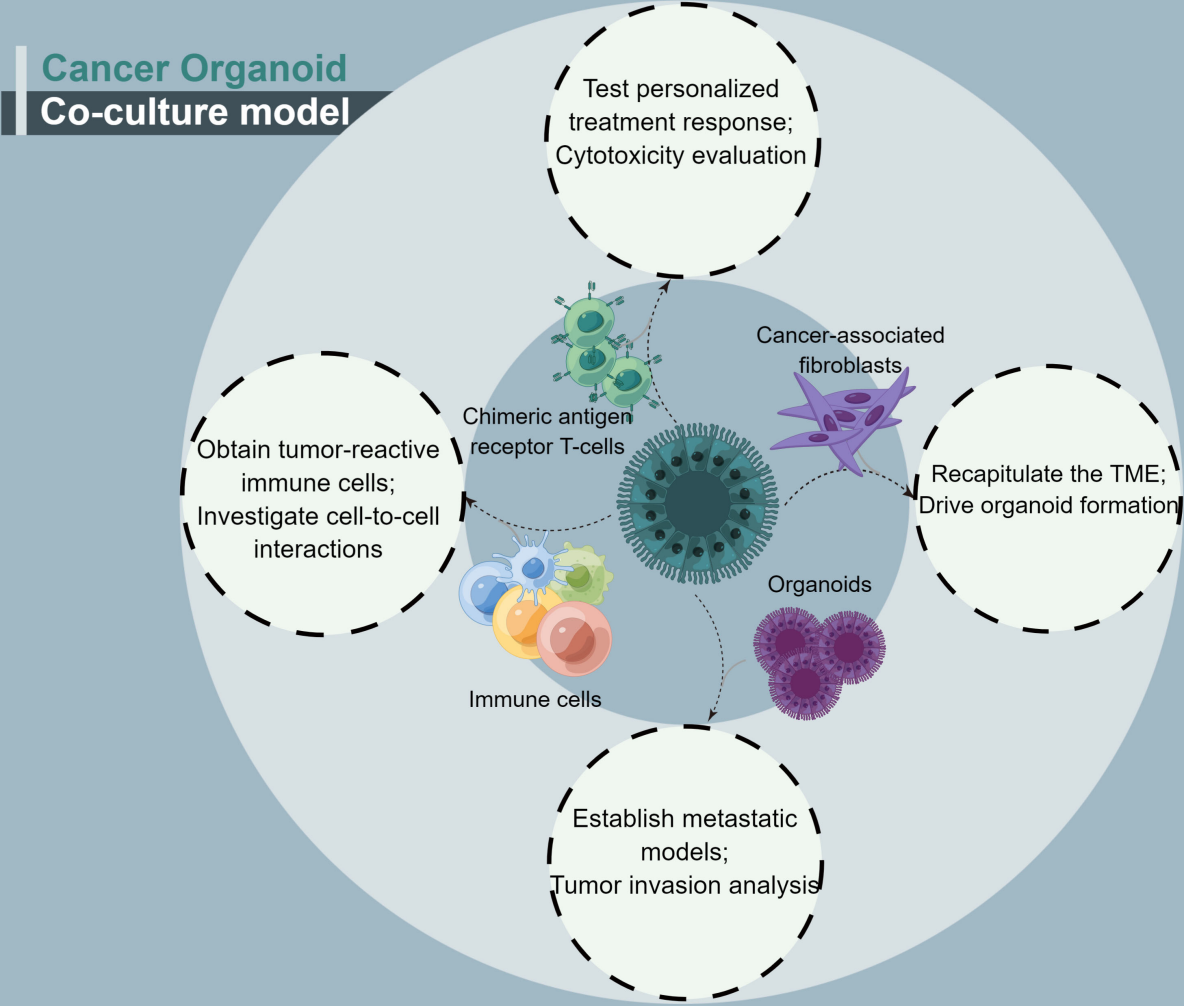
A summary of the four conditions for cancer organoids co-culture.

Cancer organoids have a major shortcoming: fewer immune cells and specific types of cancer-associated stromal cell organoids. A cancer organoid co-culture model system appears to address this issue (**Figure 3**). This approach may be considered closer to what occurs within the TME (48). With regard to the co-culture of cancer-associated cells, CAFs account for most cancer organoid co-culture studies. CAFs, as essential complements of TME, have been shown to facilitate cancer progression and promote treatment resistance. CAFs contribute to treatment resistance, mainly through impaired drug delivery and biochemical signaling (61).

Given the existence of direct or indirect biochemical crosstalk between cancer cells and CAFs, the roles of CAFs in the immune response, drug resistance, and cancer proliferation must be demonstrated in specific cancer types. Although other cell types, such as BMSCs, were observed to be radiation protective through their well-known regenerative functions after ionizing radiation (65), such co-culture models with additional conditions are encouraged in cancer research for comprehensive treatment of cancer owing to the characteristics of the subtype. Co-culture of cancer organoids and immune cells can not only establish a model system to interrogate cancer sensitivity to immunotherapy for individuals at any period during treatment but also provide a clinically practical method for the creation of patient-specific T cell products for adoptive T cell transfer through expanding circulating tumor-reactive T cells by co-culture (47). NK cells, T cells, and dendritic cells were co-cultured with cancer organoids to investigate immune responses in cancer. This novel model was considered an efficient method for rapidly evaluating the effect of immune checkpoint inhibitors on activating cytotoxic lymphocytes and increasing infiltration in the context of T-cell infiltrates (46). Single-cell T-cell receptor sequencing (scTCR-seq) is another novel single-cell approach that can identify paired α- and β-TCR subunits that determine the specificity of infiltrating T cells. Association analysis of scTCR-seq with T-cell phenotypes (activation, memory, and exhaustion) and antigen specificity determination may provide more in-depth insights into cancer immunotherapy based on this novel tool.

## 7 Conclusions

The advanced technology of co-culturing CAR-engineered lymphocytes and cancer organoids is superior in personalized medicine owing to the maintenance of heterogeneity and the TME. CAR-engineered lymphocytes combined with organoid applications in drug tests, genome editing, and high-throughput screening will be our future research direction. However, because the therapeutic effect of CAR-T cell immunotherapy in solid tumors has not been as effective as in blood cancers owing to poor trafficking, limited persistence, limited infiltration, and T cell inhibitory activity in the TME, there has been little research on this area to date (78–80). Consequently, it has been proposed that checkpoint blockade inhibitors combined with CAR-engineered cells are a promising treatment approach for solid tumors (81–85), with a platform of organoids providing recapitulation of the environment. Recently, CAR-engineered cell therapy has been expanded to novel cell types, and the expression of CARs in NK cells has been considered a more successful variant. Cancer organoid-based co-culture systems may provide a platform for evaluating the clinical therapeutic effects of adoptive cell therapies. Moreover, genetic mutations in altered surface antigens in specific cancer cells must be identified to provide primary evidence for this methodology to achieve higher targeting and therapeutic efficiency. To broaden its applications, further research is needed to establish various co-culture model systems for organoid cancer research.

## Author contributions

JY: Conceptualization, Investigation, Writing- Original draft preparation, SY, XL: Conceptualization, Supervision, Writing- Review and Editing. All authors contributed to the article and approved the submitted version.
